# Identification of novel adenovirus genotype 90 in children from Bangladesh

**DOI:** 10.1099/mgen.0.000221

**Published:** 2018-09-24

**Authors:** Charlotte J. Houldcroft, Mathew A. Beale, Md Abu Sayeed, Firdausi Qadri, Gordon Dougan, Ankur Mutreja

**Affiliations:** ^1^​ Department of Medicine, University of Cambridge, Cambridge, UK; ^2^​ Wellcome Sanger Institute, Hinxton, Cambridgeshire, UK; ^3^​ International Centre for Diarrhoeal Disease Research, Dhaka, Bangladesh

**Keywords:** adenovirus, DNA virus, metagenomics, diarrhoeal disease, *de novo* assembly, whole-genome sequencing

## Abstract

Novel adenovirus genotypes are associated with outbreaks of disease, such as acute gastroenteritis, renal disease, upper respiratory tract infection and keratoconjunctivitis. Here, we identify novel and variant adenovirus genotypes in children coinfected with enterotoxigenic *Escherichia coli*, in Bangladesh. Metagenomic sequencing of stool was performed and whole adenovirus genomes were extracted. A novel species D virus, designated genotype 90 (P33H27F67) was identified, and the partial genome of a putative recombinant species B virus was recovered. Furthermore, the enteric types HAdV-A61 and HAdV-A40 were found in stool specimens. Knowledge of the diversity of adenovirus genomes circulating worldwide, especially in low-income countries where the burden of disease is high, will be required to ensure that future vaccination strategies cover the diversity of adenovirus strains associated with disease.

## Data Summary

The genome sequence of adenovirus genotype 90 has been deposited in GenBank under accession number MH574893. Novel adenovirus genome assemblies are available from the European Nucleotide Archive, study accession PRJEB27231.

**Impact Statement**

Bangladesh, India and Pakistan bear a heavy burden of diarrhoeal disease, caused by bacteria, viruses and parasites, and the relative diversity of viral pathogens contributing to the pathogenesis of acute gastroenteritis is not fully appreciated. Our work describes the identification of novel and rare adenovirus genotypes, associated with acute gastroenteritis in children in Bangladesh, using metagenomic sequencing of stool samples. This study shows that, even in a sample of 15 children, four different adenovirus genotypes were detected in 5 patients. This suggests that any efforts to vaccinate against adenovirus and reduce the burden of gastroenteritis caused by adenovirus will need to be universal rather than genotype-specific. We also highlight the need to better characterize adenovirus diversity outside Europe and North America, which builds on recent work by other groups to better characterize circulating adenovirus diversity.

## Introduction

Adenoviruses are opportunistic human pathogens. Their dsDNA genomes are 34–36 kb in length, and the reported adenovirus genotypes (currently more than 80) can be grouped into seven species, A–G [1]. Mastadenovirus species D contains half of the reported human adenovirus (HAdV) genotypes and they are often isolated from cases of acute gastroenteritis [2–4], either as single infections or co-infections [5]. Many new adenovirus genotypes were initially identified in immune-compromised patients, such as people with AIDS [6], but adenoviruses are increasingly recognized as causing mucosal disease in immune-competent individuals, ranging from diarrhoea and keratoconjunctivitis to renal infection and potentially fatal upper respiratory tract infections [7]. No vaccine is currently available to the general population [8], while the US military vaccinates against only genotypes E4 and B7 [9].

The major capsid proteins (hexon, fibre and penton) vary within and between adenovirus species [10]. These three proteins form the major antigenic region of the virus (hexon) and play a key role in cell entry (fibre and penton) [11]. The serum neutralization properties of these three proteins have traditionally been used for the serotyping of adenovirus isolates, but new genotypes are increasingly recognized by PCR-based molecular genotyping of these regions and by whole-genome sequencing [12]. Monitoring the emergence of novel (including recombinant) genotypes is important, as it can be associated with immune escape from protection offered by natural exposure to other genotypes [13]. Whole-genome shotgun metagenomic sequencing of human clinical samples presents a valuable opportunity to recognize the emergence of new pathogens [14, 15].

Here, we report mastadenovirus genomes sequenced directly from the stool of children with enterotoxigenic *Escherichia coli* (ETEC) infection: a novel recombinant adenovirus D genome with similarities to HAdV-67 and with a genotype of P33H27F67, designated genotype 90; two A61 genomes that are closely related (99 % identity) to the Japanese A61 reference sequence; and a new adenovirus F40 genome. We also report a partial putative B16-B14 recombinant genome from a fifth child.

## Methods

### Ethics

This study utilized residual diagnostic samples collected as part of the ETEC ETVAX Vaccine Trial in Bangladesh (ETVAX/dmLT) (clinical trial NCT02531802), and was approved by the International Centre for Diarrheal Disease Research, Bangladesh (icddr,b).

### Samples

Forty stool samples were collected for this study during the recruitment of volunteers for the ETEC vaccine trial. All 40 samples were from those volunteers who did not pass the trial inclusion criteria and were eventually excluded, as they tested culture positive for ETEC when laboratory tests were performed. Ten samples each were collected from symptomatic adults (SA), asymptomatic adults (ASA), symptomatic children (SC) and asymptomatic children (ASC) at icddr,b, in 2017.

### Sequencing

Total stool DNA was extracted following a previously established protocol [16]. All extracted DNA samples were checked for quality and quantity (volume and DNA concentration) and 32 were found suitable for making sequencing libraries. Eight SC, seven SA, seven ASC and ten ASA DNA samples cleared the quality-control check and were sequenced in 16-plex on two lanes of a HiSeqV4 machine at the Wellcome Sanger Institute (WSI). Over 15 million 125 bp paired-end reads were generated for each DNA sample and the read sets were screened using a three-step process to identify (1), map (2) and (if necessary) *de novo* assemble (3) adenovirus sequences in this study as such.

#### Metagenomic analysis

Kraken [17] (default settings) was used to classify sequencing reads using a customized and comprehensive database of bacteria, viruses (dsDNA), archaea and plasmids from RefSeq, and also human and mouse references. Data was further normalized for each sample category and analysed at the phylum level. Reads assigned to mastadenovirus (species A-G) at a frequency of greater than 0.01 % of total reads were considered adenovirus positive.

#### Reference-based mapping

For each sample, sequence reads were mapped to the reference sequence for each adenovirus species using bwa [18] (0.7.17) to create a bam file. A consensus sequence was then extracted by using samtools [19] (version 0.1.19) and bcftools [20] (version 0.1.19) to call variants and generate a consensus pseudosequence, then remapped (as before) to the closest whole genome in GenBank, as identified by blastn (nucleotide collection nr/nt; somewhat similar sequences) search [21] of the consensus created by mapping to the reference sequence. The mapping parameters were default except for: the value of i was set to three times the mean fragment size as calculated from the WSI internal DNA mapping pipeline (npg). The minimum sequence depth for a base to be called was eight.

#### *De novo* assembly

For putative novel recombinant genomes, mastadenovirus-specific reads were identified and extracted using Kraken, followed by *de novo* assembly using metaSPAdes [22], with default settings. Contigs greater than 1 kb were aligned using mafft v7 (with default settings and missing bases treated as wildcards) [23], and manually merged to the reference-based assembly that recovered the greatest proportion of the genome to create a draft genome; total reads were then remapped to the draft genome using bwa as above to create a final assembly from which the consensus sequence was extracted.

### Phylogenetic analysis

Consensus genome sequences were aligned with mafft (with default settings and missing bases treated as wildcards) [23]. Maximum-likelihood phylogenies (Tamura–Nei model, 500 bootstraps) for species A and F were computed using mega7 [24]. Maximum-likelihood phylogenies for species B and D were made using raxml-ng, using the following parameters: raxml-ng –msa sequenceAlignment.fas –model GTR+FO+G –opt-branches on –opt-model on –tree pars{10}, rand{10} –all –bs-trees 100 –force –threads 2 –prefix result. The mol% G+C content was calculated using mega7. Pairwise differences and Hamming dis-similarity were computed in ugene [25], ignoring gaps and Ns.

### Recombination analysis

Recombination detection of the multiple sequence alignments was performed using Simplot (v3.5.1) for bootscanning [26]. The following parameters were used for whole-genome analysis: window size 1000, step size 200, gap stripping turned on and Kimura (two parameter) distance correction.

### Minority variant detection

Minority variants were analysed using VarScan 2.3.9 [27]. The following options were used for quality control of variant calls: –min-avg-qual 20, –min-coverage 10, –min-reads 25, –min-var-freq 0.05, –*P* value 0.01.

## Results

Mastadenovirus reads were found in samples from 5/15 children with laboratory confirmed ETEC infection. Mapping statistics, the closest reference match and predicted genotype are reported in Table 1.

**Table 1.** Mapping statistics, closest reference matches and predicted genotypes

**Sample name**	**Kraken assigned reads (%)**	**Mapping reference**	**Genotype**	**Genome recovered at 10× (%)**	**Genome recovered at 5× (%)**	**Mean depth**	**Genome length (bp)**
SC_1	0.04	KU162869	F40	99.1	99.9	41.6	34 210
SC_4	0.77	JF964962	A61	99.3	99.3	2732.1	33 777
SC_5	0.27	JF964962	A61	99.3	99.3	993.5	33 777
SC_10	0.04	AP012310 and *de novo* assembly	D90 (novel)	98.9	99.4	55.9	34 718
ASC_21	0.01	NC_011203 and *de novo* assembly	B16 or B21	76.8	97.5	12.7	35 456

Reads were mapped to known reference sequences for each species to create draft consensus sequences, identifying that two patients were infected with adenovirus of the same genotype, while the remaining three patients were infected with genotypes not shared with other patients in this small cohort. For patients SC_10 and ASC_21, no single reference genome mapping gave whole genome coverage, so *de novo* assembly with metaSPAdes was used to recover missing hexon, 5′ and 3′ sequences. *De novo* assembly of adenovirus-assigned reads (Kraken analysis) from SC_10 gave two overlapping contigs covering unique regions of the genome: 23 062 and 9408 bp in length. Following alignment, three smaller contigs between 1500 and 2500 bp in length had identical sequences to larger contigs and did not cover additional regions of the genome so were not used for further analysis. ASC_21 generated two contigs over 1000 bp: 31 660 and 2356 bp. These contigs were merged with the closest reference-based consensus to create draft genome sequences for SC_10 and ASC_21.

**Fig. 1.**
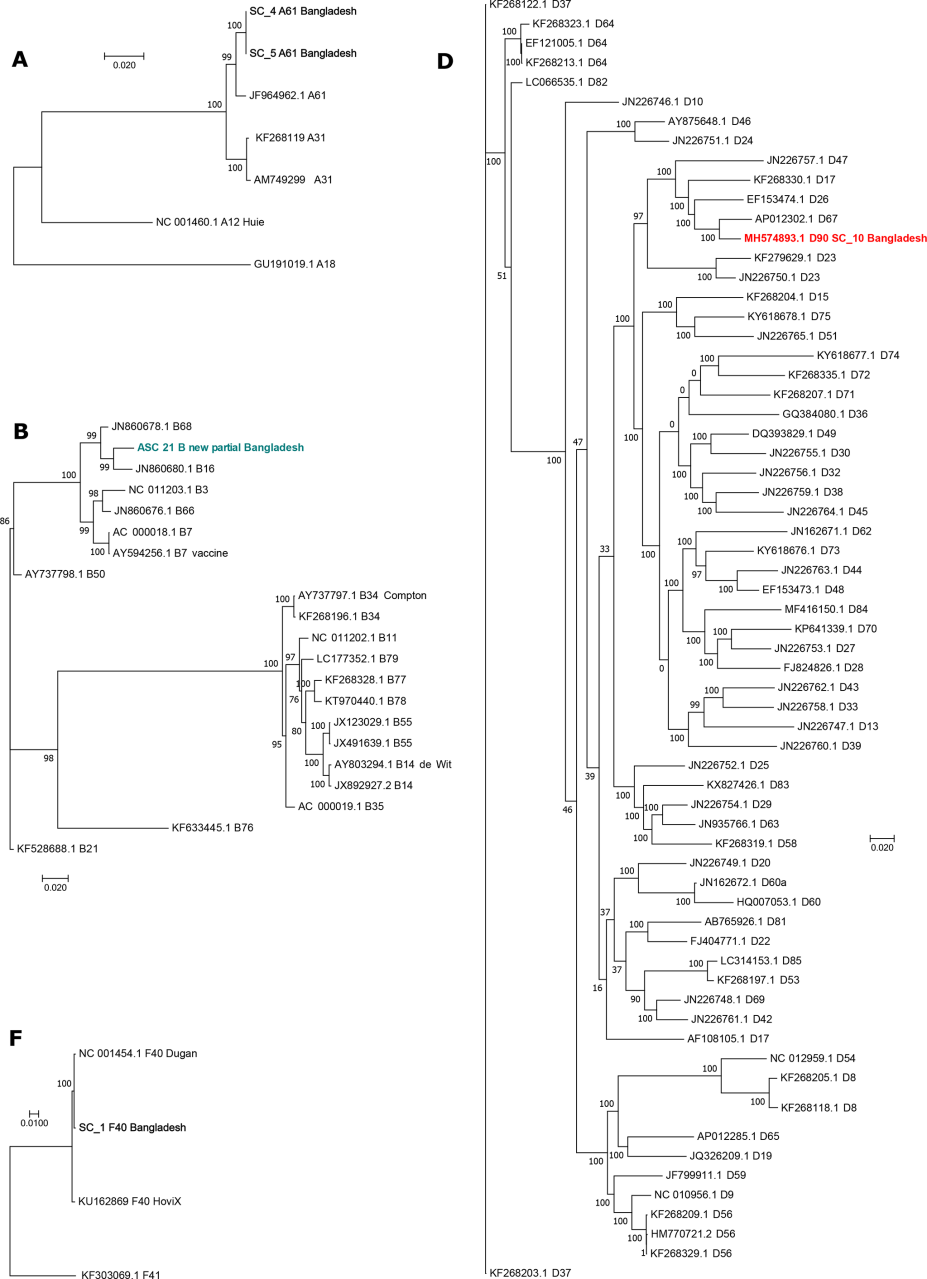
Whole-genome maximum likelihood phylogenies (Tamura–Nei model, 500 bootstraps) of adenovirus sequences identified in this study, belonging to species A, B, D and F. Novel sequences are marked in bold. The scale bar represents the number of substitutions per site.

Total reads were then remapped to the draft to generate a final genome with the genotype P33H27F67 (species D, genotype 90) and a G+C content of 57.2 mol% for patient SC_10, and a partial genome with the genotype P68H14F16 (species B) for patient ASC_21. Fig. 1 shows a whole-genome phylogeny for the consensus sequence; Fig. S1 (available with the online version of this article) shows individual phylogenetic trees of the penton, hexon and fibre genes for patients SC_10 (Fig. S1a) and ASC_21 (Fig. S1b), reflecting the recombinant nature of these genomes. SC_10 was 98 % similar to adenovirus D67, also from Bangladesh. Similarity plot (Fig. S2a) and bootscan analysis (Fig. S2b) support our hypothesis that SC_10 is a novel recombinant. SC_10 and D67 (AP012302.1) differ at 702 single nucleotide polymorphisms (SNPs), shown in Fig. S3. ASC_21 was infected with genotype B16, with some evidence of recombination with genotype B14. blastn analysis showed that the ASC_21 penton had 95 % identity to both JN860678.1 B68 (score 2787) and AY601636.1 B16 (score 2782). The most similar hexon sequences were all from genotype B14 adenoviruses, with 93 % identity to AY803294.1 (score 4248). The fibre sequence showed 99 % identity to AY601636.1 B16 (score 1505) and JN860678.1 B68 (score 1501), and 98 % identity to FJ025910.1 simian adenovirus 35.2 (score 1487) and other B16 sequences.

Previous work [28] suggests that mixed infection with two or more adenovirus genotypes may mimic a recombinant when consensus genome sequences are compared phylogenetically. These ‘consensus chimeras’ can be detected using minority variant analysis [28]. We detected 12 minority variants in sequence data from patient SC_10 at frequencies of 5 to 50 %, with 7 present at frequencies ≤20 %. These variants were distributed across the genome and were much smaller in number than the consensus level variation (SNPs) that separates D90 from closely related D67 (Fig. S3). Furthermore, the minority variants were not concentrated in areas of probable recombinant origin.

Three patients were infected with previously reported adenovirus genotypes. Patient SC_1 was infected with genotype F40. When missing bases were removed, there were 97 pairwise differences between SC_1 and Dugan [29], and 227 differences between SC_1 and Hovi X (KU162869). The SC_1 F40 sequence was >99 % similar to both publicly available F40 genomes. Patients SC_4 and SC_5 were infected with genotype A61, first reported in a case of acute gastroenteritis [30]. When missing bases were excluded, sequences from patients SC_4 and SC_5 were identical, while there were 332 SNPs separating SC_4 and the only available A61 genome sequence (JF964962). Fig. S4 shows the distribution of pairwise differences between species A consensus sequences from the same patient (data from [28]) and differences between pairs of sequences from the same genotype. The identical sequences found in SC_4 and SC_5 may indicate infection from a common source [28]. Coverage plots for each sample are shown in Fig. S5.

## Discussion

Metagenomic sequencing of stool samples from children with ETEC infection and Kraken analysis revealed, in five patients, two novel recombinant and three known adenovirus genotypes from species A, B, D and F. Using reference-based mapping and *de novo* assembly, we were able to recover whole genome sequences (>98 % genome coverage) in four cases and a partial genome (83 %) from the fifth. One patient was infected with a novel recombinant genotype of species D (P33H27F67), designated genotype 90, while another child was infected with a putative recombinant genotype of species B (P68H14F16), although only a partial genome could be recovered.

While we did not isolate the new genotype in tissue culture and thus cannot absolutely demonstrate D90’s identity as a recombinant, we are confident that the consensus sequence generated reflects the genotype infecting this patient. Minority variant analysis suggested that patient SC_10 may have been infected with a second, closely related strain of D90, present at low frequency within the stool, or a population of related D90 genomes (a viral swarm[31]). We hypothesize that these minority variants arose within the patient during the course of disease, or as the result of co-infection with two very similar D90 strains.

Homologous recombination of species D adenoviruses is widespread, while species B evolution may be driven more by base substitution [32]; however, recombination between species B genotypes can also lead to the emergence of new, virulent subtypes and disease outbreaks [33]. HAdVs have previously been described as ‘unique Trojan horse’ pathogens because of their ability to recombine, acquire new tissue tropisms and escape herd immunity [34]. As children may be persistently infected with adenovirus for months after primary infection [35], this increases the possibility of co-infection of the same host (and thus cell) with a secondary or tertiary adenovirus genotype, allowing for homologous recombination between adenoviruses of the same species [36].

Bangladesh, like many Asian and African countries, has several endemic diarrhoeal pathogens, and gastrointestinal disease cases are frequent and routine. Due to the presence of significant and hugely stigmatic pathogens such as *Vibrio cholerae* [37, 38], ETEC [39], *Shigella* sp. [40], *Salmonella typhi* [41] and rotavirus [42], the adenoviral disease burden and diversity in this region is overlooked. In our relatively small study, we were able to find new adenovirus genotypes and local variants of known genotypes, which highlights that the region is an un-tapped reservoir of novel adenoviruses that may be playing a significant and mutualistic role in local diarrhoeal outbreaks. The genotypes identified in this study are typical of adenoviruses identified elsewhere in patients with acute gastroenteritis, but quite different to those seen in adenoviraemic immune-compromised children in the UK [28]. Genotypes A61 and F40 were first described in patients with gastroenteritis [30, 43, 44]. A fourth patient was infected with a novel species D recombinant (genotype 90) that was similar to adenovirus D67 previously identified in Bangladesh [3]. One asymptomatic patient was also infected with a species B adenovirus, often associated with upper respiratory tract infections [45–47]. Metagenomic adenovirus sequencing has demonstrated particular usefulness in cases where adenovirus was not initially suspected as the causative pathogen [48] or where traditional genotyping methods have failed [49].

### Conclusion

It is unclear to what extent adenovirus contributes to the symptoms of diarrhoea as a co-pathogen [5] in Bangladesh; further study of ETEC-negative stool samples from this region will be required to address this question. Given the diversity of strains identified in this small study, metagenomic sequencing is an important tool for characterization of the role of adenovirus in diarrhoeal disease.

## Data bibliography

1. Mutreja A, Houldcroft CJ. D90 genome sequence. GenBank accession no. MH574893.1 (2018).
2. Mutreja A, Houldcroft CJ. Adenovirus genomes reported in this study. European Nucleotide Archive BioProject PRJEB27231, sample accessions ERS2541748 ERS2541752 (2018).

## Supplementary Data

Supplementary File 1
