# Supplementary material for: Identification of novel adenovirus genotype 90 in children from Bangladesh
Source: Microb Genom. 2018 Sep 24;4(10):e000221. doi: 10.1099/mgen.0.000221 (PMC6249435; doi:10.1099/mgen.0.000221)
Supplement: Supplementary File 1 [file mgen-4-221-s001.pdf]

Supplementary figure 1

Plot showing the phylogenies of the penton, hexon and fiber genes of the novel recombinant species D genotype 90 virus identified in this study. The penton clusters phylogenetically with genotype 33, the hexon with genotype 27 and the fiber with genotype 67 (all members of adenovirus species D).

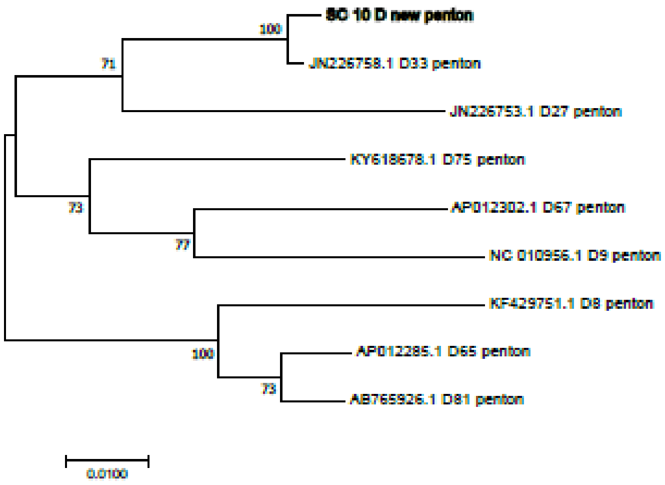
SC 10 D new penton
JN226758.1 D33 penton
JN226753.1 D27 penton
KY618678.1 D75 penton
AP012302.1 D67 penton
NC 010956.1 D9 penton
KF429751.1 D8 penton
AP012285.1 D65 penton
AB765926.1 D81 penton
100
71
73
77
100
73
0.0100

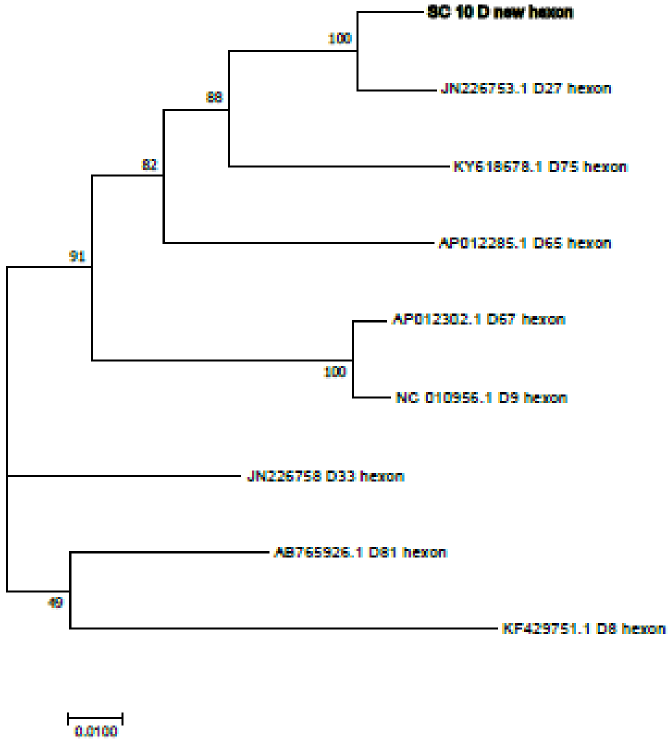
SC 10 D new hexon
JN226753.1 D27 hexon
KY618678.1 D75 hexon
AP012285.1 D65 hexon
AP012302.1 D67 hexon
NC 010956.1 D9 hexon
JN226758 D33 hexon
AB765926.1 D81 hexon
KF429751.1 D8 hexon
100
88
82
91
100
49
0.0100

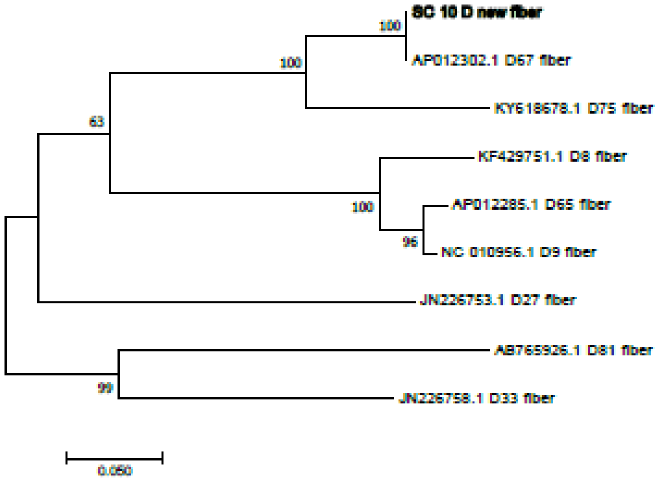
SC 10 D new fiber
AP012302.1 D67 fiber
KY618678.1 D75 fiber
KF429751.1 D8 fiber
AP012285.1 D65 fiber
NC 010956.1 D9 fiber
JN226753.1 D27 fiber
AB765926.1 D81 fiber
JN226758.1 D33 fiber
100
100
63
100
96
99
0.050

Supplementary figure 2

A. Similarity plot and Bootscan analysis of the novel recombinant adenovirus D genotype 90 reported here, compared to the most closely related species D genotypes. Both SimPlot and Bootscan analyses were performed with a window size of 1000 bp and a step size of 200bp. These plots suggest that SC_10 has a recombinant genotype with similarity to D27, D33 and D67.
B. Similarity plot and Bootscan analysis of the putative recombinant adenovirus B partial genome reported here, compared to the most closely related species B genotypes. Both analyses were performed as above. These plots suggest that ASC_21 has a complex recombinant genotype with similarity to B14, B16, B68 and genome KF528688, itself a B16/B21 recombinant.

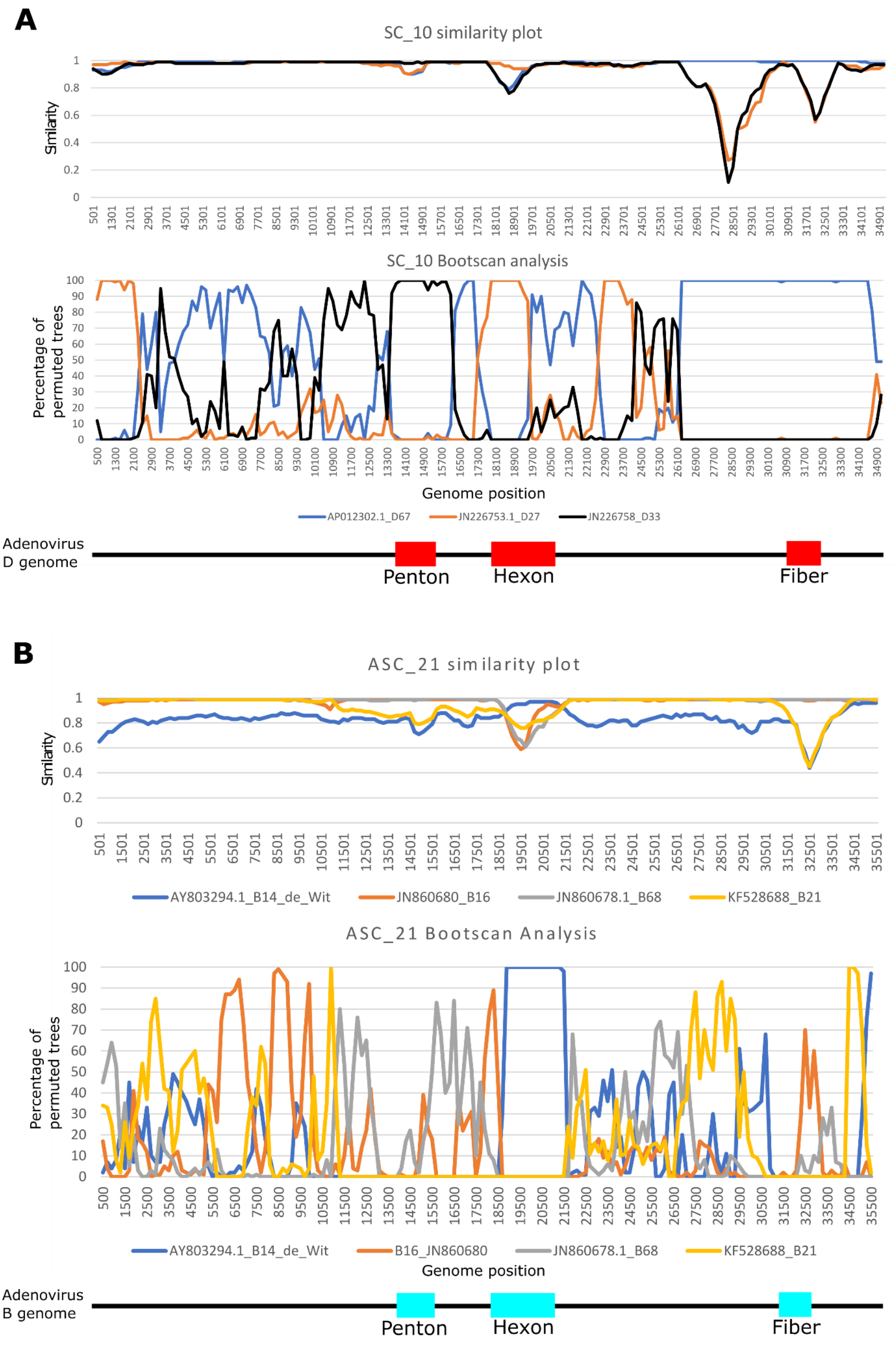

A
SC_10 similarity plot
Similarity
SC_10 Bootscan analysis
Percentage of permuted trees
Genome position
AP012302.1_D67
JN226753.1_D27
JN226758_D33
Adenovirus D genome
Penton
Hexon
Fiber
B
ASC_21 similarity plot
Similarity
AY803294.1_B14_de_Wit
JN860680_B16
JN860678.1_B68
KF528688_B21
ASC_21 Bootscan Analysis
Percentage of permuted trees
AY803294.1_B14_de_Wit
B16_JN860680
JN860678.1_B68
KF528688_B21
Genome position
Adenovirus B genome
Penton
Hexon
Fiber

Supplementary figure 3

A. Plot showing the location of consensus SNPs in the genome of SC_10 (novel adenovirus genotype D90), compared to the D67 reference sequence AP012302.1. Gaps and Ns in the sequences were ignored. Sequences were aligned using MAFFT.

B. Plot showing the location of high-confidence minority variant (sub-consensus) alleles called using VarScan2, called relative to the SC_10 consensus pseudosequence (Methods). Reference alleles are present in the consensus pseudosequence but at lower frequency than an alternate allele in the final genome mapping for SC_10, which produced the consensus sequence for genotype D90. Minority alleles are alleles which were not present in the consensus pseudosequence or the final consensus sequene, but which likely represent true within-patient diversity rather than sequencing or mapping errors. There are more between-genotype consensus variants than within-patient minority variants.

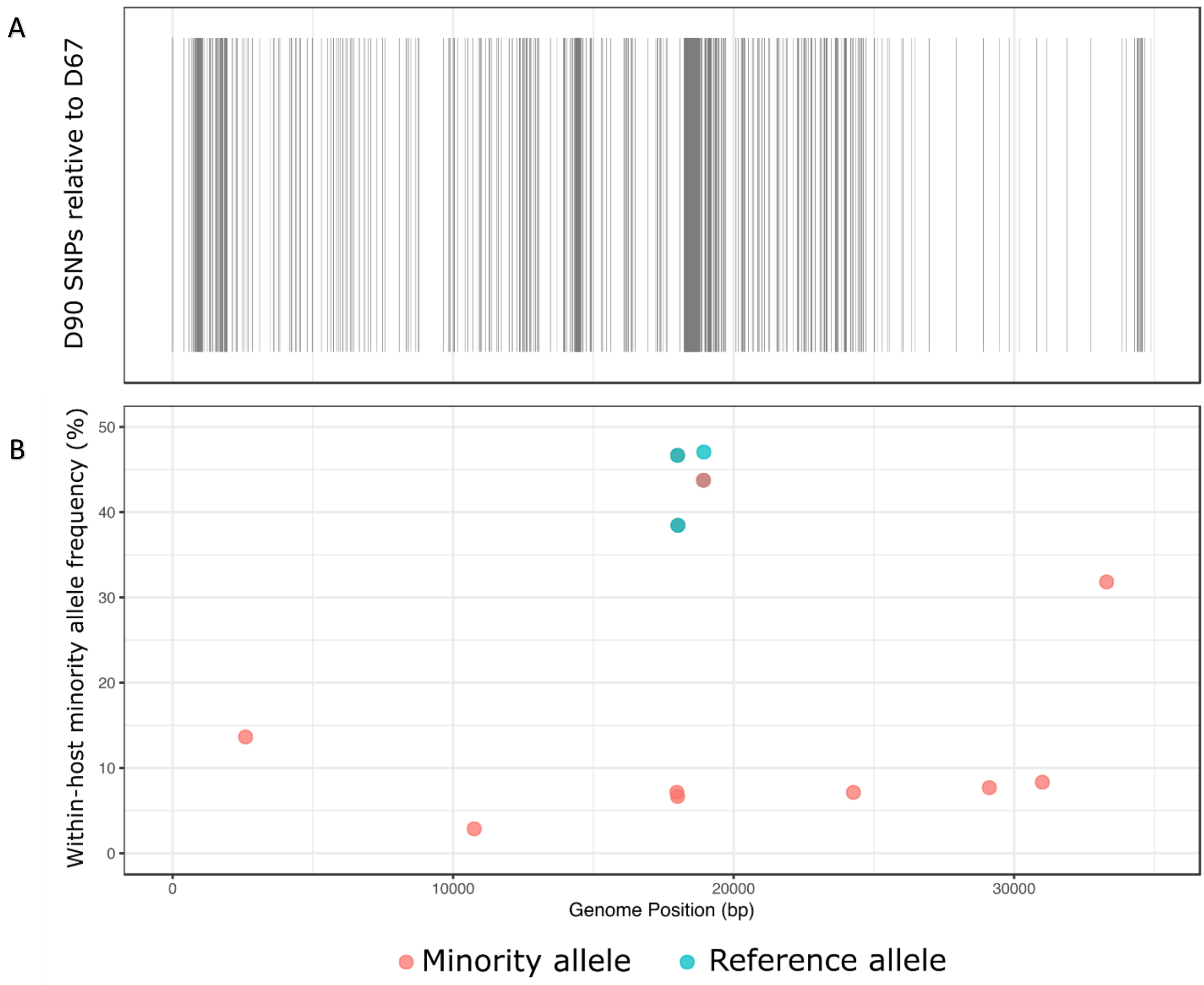
A
D90 SNPs relative to D67
B
Within-host minority allele frequency (%)
50
40
30
20
10
0
0
10000
20000
30000
Genome Position (bp)
Minority allele
Reference allele

Supplementary figure 4

Plot showing the distribution of pairwise differences between species A consensus sequences. Blue indicates pairwise differences between consensus sequences from the same patient sampled longitudinally (data from [31]). Red indicates pairwise differences between individuals infected with the same species A genotype (ie both A61 or both A31). Yellow indicates pairwise differences between different genotypes within species A (eg A12 and A18).

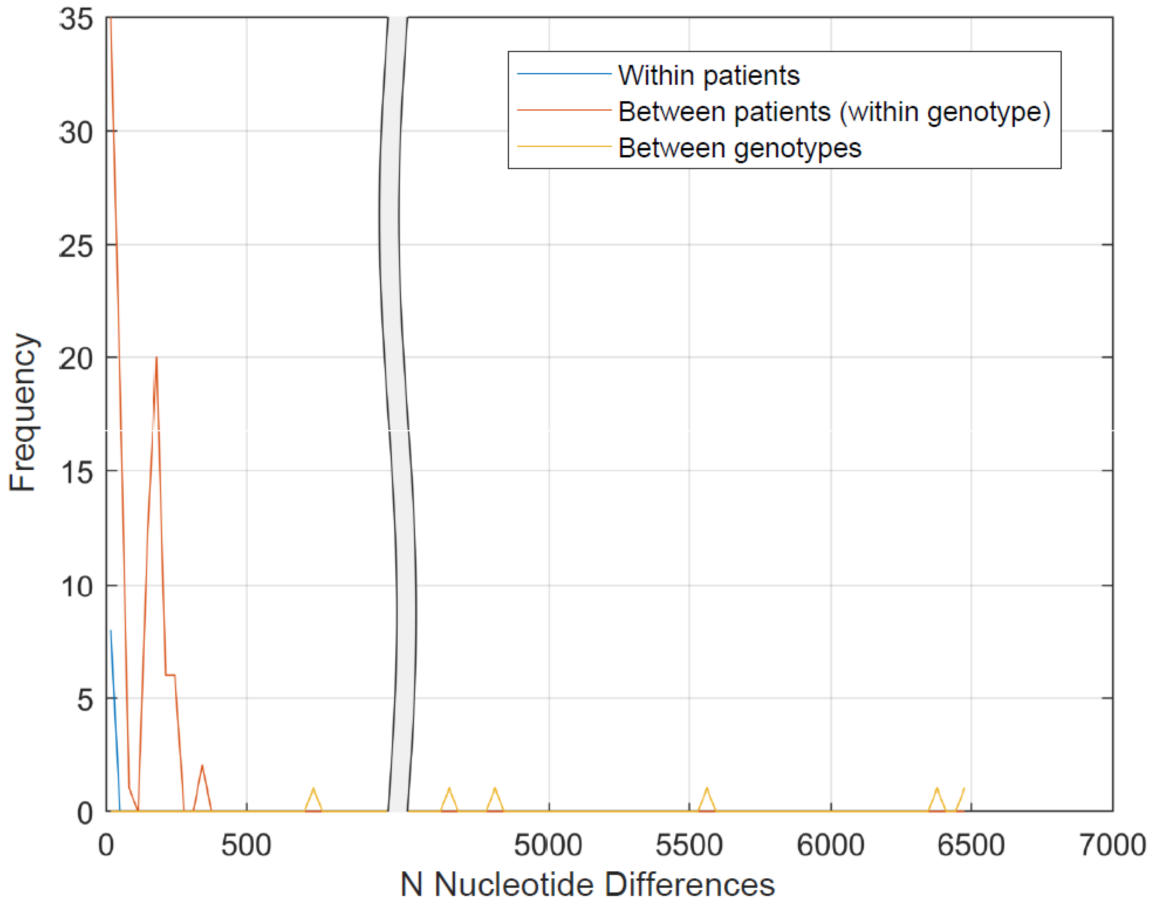

Supplementary figure 5

Genome-wide coverage statistics (reference-based mapping) for each adenovirus genome sequence assembled in this study.

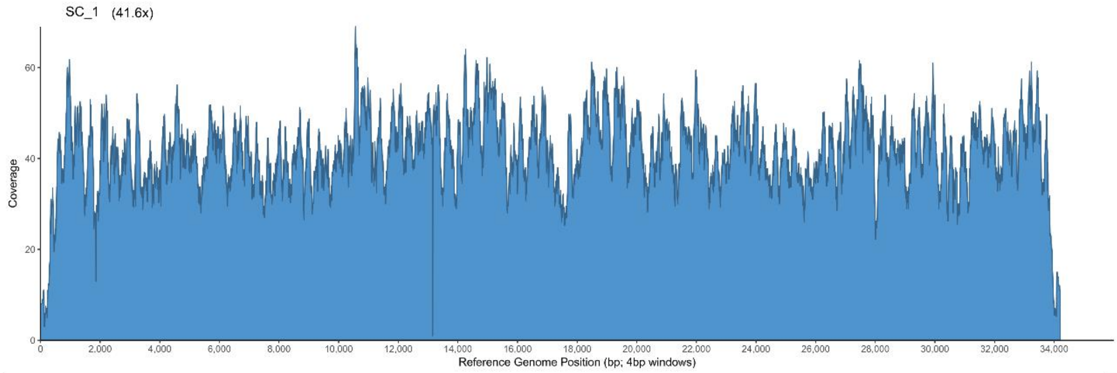
SC_1 (41.6x)
Coverage
Reference Genome Position (bp; 4bp windows)

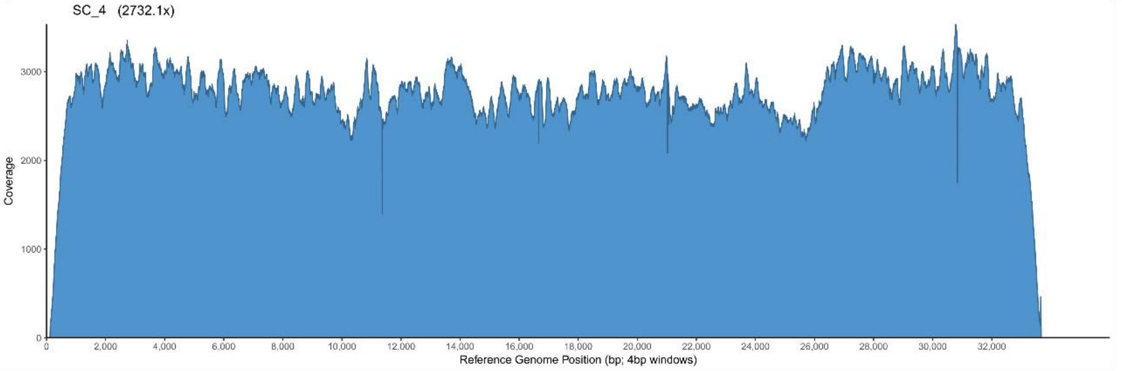
SC_4 (2732.1x)
Coverage
Reference Genome Position (bp; 4bp windows)

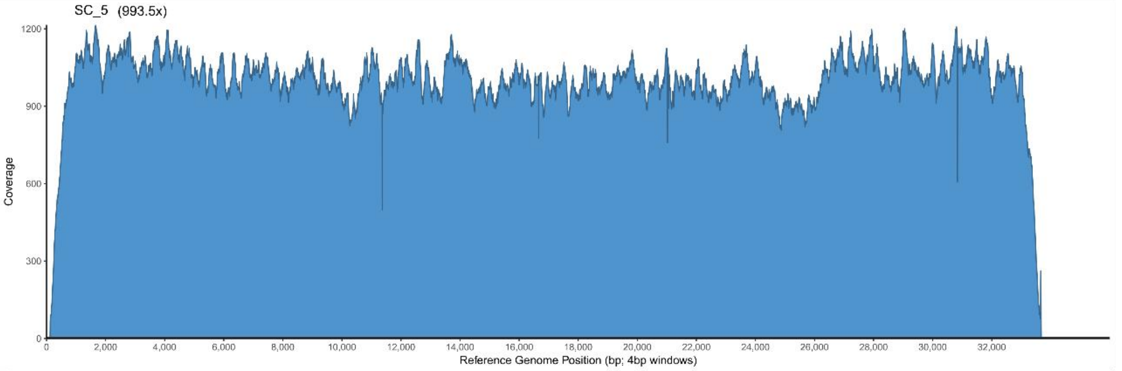
SC_5 (993.5x)
Coverage
Reference Genome Position (bp; 4bp windows)

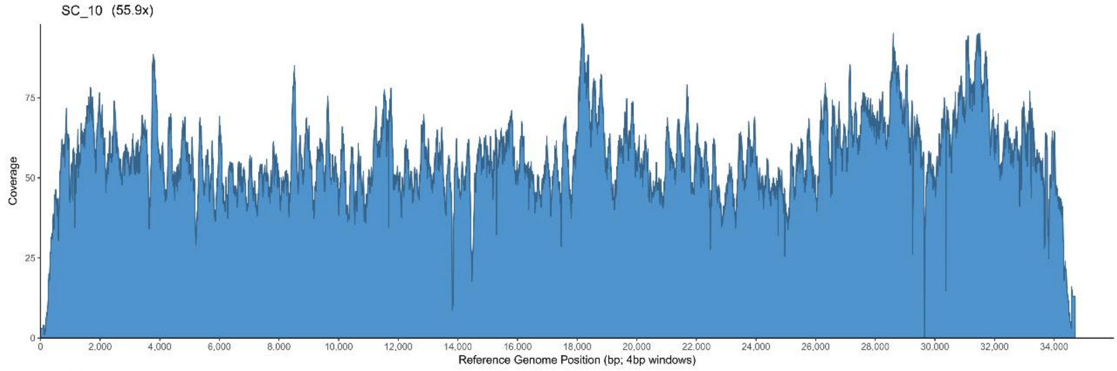
SC_10 (55.9x)
Coverage
Reference Genome Position (bp; 4bp windows)

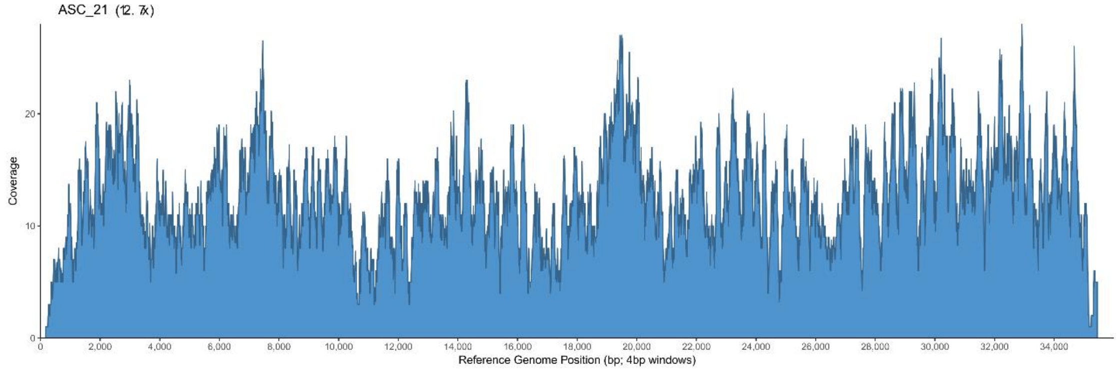
ASC_21 (12. 7x)
Coverage
Reference Genome Position (bp; 4bp windows)
